# Supplementary material for: Reporting Criteria for Clinical Trials on Medication-Related Osteonecrosis of the Jaw (MRONJ): A Review and Recommendations
Source: Cells. 2022 Dec 16;11(24):4097. doi: 10.3390/cells11244097 (PMC9777472; doi:10.3390/cells11244097)
Supplement: Supplementary file 1 [file cells-11-04097-s001.zip › cells-1883830-supplementary.pdf]

Supplementary materials

Legend for Tables 1, S1 and S2: AAOMS: Association of Oral and Maxillofacial Surgeons; BPP: Bisphosphonates; BFPF: buccal fat pad flap; CBCT: cone beam computed tomography; CT: Computer tomography; FDG: Fluorodeoxyglucose; IV: Intravenous ; mAb: monoclonal antibody; MRI: Magnetic resonance imaging; MRONJ: Medication-related osteonecrosis of the jaw; NA: Not applicable; OAC: Oroantral communication; OPT: Orthopantomography ; PET: Positron emission tomography; PRF: Plasma rich fibrin; QOL: Quality of life; RCT: Randomized clinical trial; VAS: Visual analogue scale ; VELscope: Visually Enhanced Lesion Scope; (-): Not specified.

Table S1 (part A and B). Summary of the 12 essential criteria extracted from the selected articles

Table S1 part A

References	Patient characteristics					Surgery
	Classification	Mucosal defect measurement	Drug Holiday	Number of sites affected	Number of sites treated	PRF
[57]	AAOMS stage	No	If BPP IV change for BPP per os	–	All sites	No
[74]	AAOMS stage	No	–	–	–	No

[80]	AAOMS stage	No	_	_	_	No
[13]	Necrotic bone exposure in mandible or maxilla for 3 months in patients who had received, or were receiving, either IV or oral BPP therapy	No	Yes (2 months before surgery)	_	_	No
[56]	AAOMS stage	No	Yes (2 weeks before surgery and 1 month after)	≥ 2	_	No
[49]	Marx classification	No	No, unless a break was recommended by oncologist	_	_	No
[60]	AAOMS stage	No	Yes	_	_	No
[73]	AAOMS stage	No	_	_	_	No

[59]	AAOMS stage	No	No	_	_	No
[61]	AAOMS stage	No	Yes (42%)	_	_	No
[55]	AAOMS stage	No	Yes (2 weeks before surgery and 2 weeks after)	2	_	No
[72]	AAOMS stage	No	_	2	_	Yes
[58]	AAOMS stage	No	No	_	_	No
[62]	AAOMS stage	No	_	_	_	No

[70]	AAOMS stage	No	Yes (100%)	2-3	_	No
[45]	AAOMS stage	Yes	_	_	_	No
[37]	Marx classification	No	No	_	_	Yes
[50]	Clinically with localized osteolytic bone lesions	No	_	_	_	Yes
[77]	AAOMS stage	No	Yes (12.5%)	_	_	No

[71]	AAOMS stage	No	Yes (100%)	3	_	No
[75]	AAOMS stage	No	Yes (15%)	_	_	No
[69]	AAOMS stage	No	_	2-3	_	No
[76]	AAOMS stage	No	_	_	_	No
[63]	AAOMS stage	No	Yes (31.9%)	_	_	No
[64]	AAOMS stage	No	_	_	_	No

[52]	AAOMS stage	No	_	_	_	No
[43]	AAOMS stage	No	_	_	_	No
[79]	AAOMS stage	No	Yes (100%)	_	_	No
[83]	AAOMS stage	No	Yes (100%)	2	_	No
[54]	AAOMS stage	No	Yes (> 3 months = 22 (12 surgical, 10 non-surgical), 0-3 months = 30 (16 surgical, 14 non-surgical))	_	_	No
[46]	AAOMS stage	No	Yes	_	_	No

[78]	AAOMS stage	No	_	2	_	No
[53]	AAOMS stage	No	Yes (minimum 3 months before surgery)	2	_	No
[47]	AAOMS stage	No	_	_	_	YES
[65]	AAOMS stage	No	_	_	_	No
[36]	AAOMS stage	No	No, unless a break was recommended by oncologist	> 1	_	No

[82]	AAOMS stage	No	Yes (50%)	_	_	No
[38]	AAOMS stage	No	Yes (with physician's advice)	>1	_	No
[66]	AAOMS stage	No	Yes (if oncological point of view was considered justifiable)	_	_	No
[67]	AAOMS stage	No	_	2	_	No

[68]	AAOMS stage	No	_	_	_	No
[39]	AAOMS stage	No	Yes (100%): 3 months before surgery	2	_	Yes
[51]	AAOMS stage	Yes	YES: 3 weeks before surgery: 4/5 cases	2	All sites	No

Table S1 part B

References	Study characteristics									
	Primary outcome	How primary outcome was evaluated	When primary outcome was evaluated	Secondary outcomes and evaluation	Definition of failure	Failure or healing rate	Re-exposed bone	Follow-up (months)	Imaging tools pre-intervention	Imaging tools post-intervention
[57]	Intact mucosa	Clinically	At last follow-up visit	Pain (VAS), dental rehabilitation appliances	–	–	–	6	–	OPT
[74]	Bone covered by mucosal flaps with no signs or symptoms; Healing (evaluated radiologically)	Clinically and radiologically	–	–	–	0 % (surgically), 100% (conservative)	–	39 (12-80)	Radiography	Healing was evaluated radiologically
[80]	Complete closure of mucosa	Clinically	At the review	Wound dehiscence, pain, discomfort, and oral hygiene	–	0%	Clinically	24	–	–

[13]	Complete mucosal coverage and elimination of pain	Clinically	_	_	_	15%	Clinically	1 to 40 (mean: 10.7)	CT scan	_
[56]	Oral examination without pathological findings	_	At last follow-up visit	_	MRONJ recurrence	4% in the first 2 months, 6% between 4 to 6 months	Clinically	12	CT scan	_

[49]	Intact mucosa in the operated area with no signs of infection or sinus formation, cessation of the bony destructive process or bony remodeling of the operated area	Clinically and radiologically	At the review	–	–	0%	–	6-48 (mean: 20)	Radiography	Healing was evaluated radiologically
[60]	24-month recurrence rate of MRONJ and 24-month mortality rate	Clinically	24 months	Postoperative complications, duration of hospital stay after surgery, time to return to oral feeding, and degree of oral pain	–	–	Clinically	24	OPT + CT scan	OPT + CT scan

[73]	Full epithelization of the exposed bone and no signs of infection	Clinically	At the review	_	_	60 % surgical technique	_	3-28	OPT + CT scan	_
[59]	Mucosal closure and absence of symptoms 1 month postoperatively	Clinically	1 month	_	_	15%	_	1	VELscope® system	_
[61]	Complete healing of MRONJ: no exposed necrotic bone, no residual mucosal defect, no fistulas, and absence of swelling and pain	Clinically	At the review	_	Non healing sites or recurrence of clinical signs of MRONJ (exposed bone, mucosal defect, fistulas, swelling, pain)	12%	_	15	CBCT	_
[55]	Recurrence of MRONJ	Clinically	_	Identified important prognostic factors related to recurrence	_	_	_	_	OPT	_

[72]	Absence of pain and bone exposure	_	At last follow-up visit	_	Partial response: absence of pain and presence of bone exposure / No response: persistence of pain and bone exposure	_	_	6	Imaging tests	OPT
[58]	Intact oral mucosa or no symptoms	Clinically	_	_	_	10%	Clinically	3-33	_	_
[62]	Clinical improvement and complete healing	Clinically	_	_	_	Improvement: 18.5% (non-cancer) / 37.25% (cancer); Healing: 28.5% (non-cancer) / 46.1% (cancer)	Clinically	16.44 ± 10.95 (6-54)	OTP + CT scan	_
[70]	Complete recovery of the mucosa	Clinically	At the review	_	_	4.8%	Clinically	16 (12-24)	OPT + CT scan	Radiological examinations

[45]	Healing (gingival coverage with no exposed bone), improvement (decrease in size or number of baseline lesions), unchanged (no change in size or number of baseline lesions), or worse (increase in size or number of baseline lesions compared with their condition at the time of consent)	Clinically	At the review	Pain and QOL/Duke Health Profile	–	–	–	24	–	–
[37]	Maintenance of mucosal closure without clinical and radiographic signs of residual infection or exposed bone	Clinically	–	Pain or discomfort	–	0%	Clinically	45-60	CT scan	–

	at the time of evaluation									
[50]	Absence of bone exposure and osseous lesions	Clinically	At last follow-up visit	–	–	PRF group: 0% failure, non-PRF group: 100% failure	–	3	–	X-ray
[77]	Improvement between pre- and post-operative stages of disease	Clinically	At the review	Repeat surgery	–	–	–	Mean: 11.2 (±10.6) after first visit, and 6.7 (±7.5) after surgery	–	–
[71]	Mucosa with no signs of infection or sinus formation	Clinically	At the review	–	–	–	–	7-25	–	–
[75]	Mucosal closure postoperatively over the whole follow-up period	Clinically	At last follow-up visit	–	Mucosal dehiscence within the first month postoperatively	5%	Clinically	12 (4-18)	OPT +/- CBCT +/- MRI +/- scintigraphy	–

[69]	Complete mucosal coverage without any exposed bone and without any sign of infection	Clinically	At the review	–	–	17% (osteoporosis), 74% (malignant disease)	–	Healing date + 3 months	–	–
[76]	Symptom-free condition including mucosal closure	Clinically	24 months	–	–	10% surgical treatment	Clinically	12 (stage 1)	–	–
[63]	Complete mucosal healing and lack of clinical symptoms 3 months after surgery	Clinically	3 months	–	No change in MRONJ stage 3 months after surgery or MRONJ stage III was only downscaled to MRONJ stage II	25%	–	3	CT scan	–
[64]	Postoperative healing: mucosal closure without signs of infection or exposed bone	Clinically	At the review	–	–	A (low risk) 0% / B (high risk): 73%	–	6.6 (2-48)	CT scan or radiographs and PET-CT (identify lesions) + FDG (surgery choice)	–

[52]	(i) maintenance of mucosal closure without signs of infection or exposed bone at the time of evaluation, (ii) decrease in MRONJ stage if the first endpoint was not achieved	Clinically	At the review	–	–	72.4%	–	13.7 (6-24)	–	–
[43]	Full mucosal coverage without signs of residual infection or exposed bone at the time of last follow-up	Clinically	At the last follow-up visit	–	–	3/54 patients (5.6%)-3/65 lesions (4.6%)	–	12.9 (median: 11) (1-39)	VELscope® system	–
[79]	Condition with no complaints, and healed or closed mucosa	Clinically	At the review	–	–	6.8 % (6 months)	–	> 6 (6-96)	–	–
[83]	Healed and closed mucosa without complaints	Clinically	1 month	–	–	–	–	6-34 (mean: 16.4)	–	–

[54]	Complete disappearance of exposed bone without clinical symptoms	Clinically	6 months	–	Bone remains exposed or disease progression	Surgical treatment: 11% / No surgical treatment: 67%	–	6	–	–
[46]	Complete healing: complete regrowth of oral mucosa over exposed bone, for at least 3 months	Clinically	> 3 months	–	–	Surgical treatment: partial healing 18% / Stable disease 4% / Progression 0.6% (3 months)	–	15 (3-81)	–	–
[78]	No exposed bone 2 months after surgery	Clinically	2 months	Mucosal integrity, signs of infection, pain, and loss of sensitivity/ clinically	–	25%	–	12	VELscope® system	–
[53]	Complete mucosal coverage with absence of MRONJ signs and symptoms	Clinically	–	–	–	30%	–	10.4	–	–

[47]	Mucosal integrity 6 months after surgery	Clinically	6 months	Absence of infection, signs of fistula, re-intervention, pain (VAS)	–	–	Clinically	12	OPT +/- CBCT	–
[65]	Mucosal integrity 6 months after surgery	Clinically	6 months	Mucosal integrity at the remaining follow-up visits and no signs of residual infection at T1, T2, T3, and T4	–	–	Clinically	12	OPT +/- CBCT	–
[36]	No bone exposure and no infection in the jaws 2 months after treatment	Clinically	2 months	Pain (VAS)	–	–	–	2-46	–	–
[82]	Successful wound closure defined as a symptom-free and closed wound after at least 12 months	Clinically	> 12 months	–	–	–	–	≥ 12 (15-17)	–	–

[38]	Complete healing of MRONJ: no exposed necrotic bone, no residual mucosal defect, no fistulas and absence of swelling and pain	Clinically	> 24 months	–	Re-intervention	Stage 2: 13%, Stage 3: 43%	Clinically	24	CT scan	OPT
[66]	Mucosal integrity (maintenance of full mucosal coverage after surgery without signs of residual infection or exposed bone at the review)	Clinically	8 months	–	–	9.2% (8 months)	Clinically	8	–	–
[67]	Complete epithelialization of surgical site without inflammation and pain after single-layer closure with mucoperiosteal flap and double-layer	Clinically	–	No signs of infection and absence of disease	–	–	–	3 to 48 (mean: 6)	CT scan	–

	closure with BFPF for MRONJ lesions with or without OAC									
[68]	(i) complete healing, defined as complete coverage of previously exposed bone by oral mucosa; (ii) partial healing, defined as lesion downstaging according to AAOMS criteria; (iii) stable disease, defined as no change in the lesion's clinical stage during observation; and (iv) progressive	Clinically	–	–	–	–	–	–	CT scan	CT scan

	disease, defined as lesion upstaging during observation										
[39]	Successful surgical treatment (hard and soft tissue healed at treated site, disappearance of any symptoms) and the occurrence of postsurgical complications	Clinically	At the review	–	–	0%	–	23.5 +/- 8.7 (12–36)	OPT +/- CT scan	X-ray at 3 years
[51]	Improvement: total mucosal coverage, no sign of infection and no pain	Clinically and VAS for pain	3 months	–	No improvement	0%	–	3	–	OPT + CT scan

Table S2. Summary of seven additional items

References	Study		Patient characteristics			Treatment strategy	
	RCT	Number of patients / Number of lesions	Medication	MRONJ grade / Site of lesion if indicated	Number of patients with multiple lesions	Grade of MRONJ treated	Treatment differs by stage
[57]	No	58	BPP per os or IV	1-3	10	All grades	Yes
[74]	No	49	BPP	1-3	_	All grades	No
[80]	No	15	BPP per os or IV	1	1	Only grade 1	No

[13]	No	33	BPP per os or IV	NA	4	All grades	No
[56]	No	50	BPP IV	1-3	1	All grades	No
[49]	No	40	BPP	(_) or Marx classification (?)	_	_	No
[60]	No	30 (32 lesions)	BPP per os or IV	0-3	_	All grades	No
[73]	No	20	BPP IV	1-2	_	All grades	No
[59]	No	15 (20 lesions)	BPP IV	2-3	2	All grades	No

[61]	No	24 (33 lesions)	BPP IV	1-3	_	All grades	No
[55]	No	108	BPP per os or IV	1-3	7	All grades	Yes
[72]	No	22	BPP per os or IV	1-3	2	_	_
[58]	No	20	BPP per os or IV	1-2	_	All grades	No
[62]	No	190 (166 sites)	BPP	1-3	_	All grades	No
[70]	No	21	BPP per os or IV	2-3	2	All grades	No

[45]	Yes	49	BPP per os or IV	1-3	_	All grades	No
[37]	No	32	BPP IV	Marx IIB classification	_	Only lesions meeting Marx IIB classification	NA
[50]	No	7	BPP IV	NA	_	_	_
[77]	No	88	BPP per os or IV	1-3	_	All grades	Yes
[71]	No	11	BPP IV	2-3	2	All grades	No

[75]	No	20	BPP per os or IV	1-3	3	All grades	No
[69]	No	55	BPP per os or IV	1-3	4	All grades	Yes
[76]	No	38	BPP per os or IV	0-3	_	All grades	Yes
[63]	No	39 (47 lesions)	BPP IV	2-3	_	All grades	No
[64]	No	31 (33 lesions)	BPP, Denosumab	2-3	_	All grades	No

[52]	No	40	BPP per os, IV, Denosumab	1-3	_	All grades	No
[43]	No	54 (65 lesions)	BPP, Denosumab	0-3	_	All grades	No
[79]	No	74	BPP per os or IV	2-3	5	All grades	No
[83]	No	11	Denosumab	2-3	1	All grades	No
[54]	No	52	BPP IV and per os, Denosumab	2	_	Only grade 2	NA
[46]	No	361	BPP IV, Denosumab	1-3	_	All grades	No

[78]	Yes	40	BPP, Denosumab	0-3	1	_	_
[53]	No	63 (69 lesions)	Denosumab (+/- history of BPP in 49%)	0-3	_	Only grade 2	NA
[47]	Yes	47 (61 lesions)	BPP IV or per os, Denosumab	2-3	_	Grade 2, 3	_
[65]	Yes	36 (39 lesions)	BPP, Denosumab	1-3	_	_	_
[36]	No	55	BPP IV or per os, Denosumab	0-3	5	_	No

[82]	No	32	BPP or mAb	1-3	_	_	No
[38]	No	53	BPP per os	1-3	2	_	No
[66]	No	87 (104 lesions)	BPP, Denosumab	1-3	_	All grades in each group	No
[67]	No	72 (79 lesions)	BPP, Denosumab	2-3	_	_	_

[68]	No	106 (131 lesions)	BPP, Denosumab	1-3	–	–	–
[39]	No	11	BPP per os (alendronate)	2	8	Only grade 2	NA
[51]	No	5	BPP IV	2	2	Only grade 2	NA
